# Delayed consultation among pulmonary tuberculosis patients: a cross sectional study of 10 DOTS districts of Ethiopia

**DOI:** 10.1186/1471-2458-9-53

**Published:** 2009-02-09

**Authors:** Mengiste M Mesfin, James N Newell, John D Walley, Amanuel Gessessew, Richard J Madeley

**Affiliations:** 1 Nuffield Centre for International Health and Development, Institute of Health Sciences, University of Leeds, Leeds, UK; 2 Mekelle University Medical College, Mekelle, Ethiopia; 3 University of Nottingham Medical School, Public Health Medicine and Epidemiology, School of Community Health Science, Nottingham, UK

## Abstract

**Background:**

Delays seeking care increase transmission of pulmonary tuberculosis and hence the burden of tuberculosis, which remains high in developing countries. This study investigates patterns of health seeking behavior and determines risk factors for delayed patient consultation at public health facilities in 10 districts of Ethiopia.

**Methods:**

New pulmonary TB patients ≥ 15 years old were recruited at 18 diagnostic centres. Patients were asked about their health care seeking behaviour and the time from onset of symptoms to first consultation at a public health facility. First consultation at a public health facility 30 days or longer after onset of symptoms was regarded as prolonged patient delay.

**Results:**

Interviews were held with 924 pulmonary patients. Of these, 537 (58%) were smear positive and 387 (42%) were smear negative; 413 (45%) were female; 451 (49%) were rural residents; and the median age was 34 years. Prior to their first consultation at a public health facility, patients received treatment from a variety of informal sources: the Orthodox Church, where they were treated with holy water (24%); private practitioners (13%); rural drug vendors (7%); and traditional healers (3%). The overall median patient delay was 30 days (mean = 60 days). Fifty three percent [95% Confidence Intervals (CI) (50%, 56%)] of patients had delayed their first consultation for ≥ 30 days. Patient delay for women was 54%; 95% CI (54%, 58%) and men 51%; 95% CI (47%, 55%). The delay was higher for patients who used informal treatment (median 31 days) than those who did not (15 days). Prolonged patient delay (≥ 30 days) was significantly associated with both patient-related and treatment-related factors. Significant patient-related factors were smear positive pulmonary disease [Adjusted Odds Ratio (AOR) 1.4; 95% CI (1.1 to 1.9)], rural residence [AOR 1.4; 95% CI (1.1 to 1.9)], illiteracy [AOR 1.7; 95% CI (1.2 to 2.4)], and lack of awareness/misperceptions of causes of pulmonary TB. Significant informal treatment-related factors were prior treatment with holy water [AOR 3.5; 95% CI (2.4 to 5)], treatment by private practitioners [AOR 1.7; 95% CI (1.1 to 2.6)] and treatment by drug vendors [AOR 1.9; 95% CI (1.1 to 3.5)].

**Conclusion:**

Nearly half of pulmonary tuberculosis patients delayed seeking health care at a public health facility while getting treatment from informal sources. The involvement of religious institutions and private practitioners in early referral of patients with pulmonary symptoms and creating public awareness about tuberculosis could help reduce delays in starting modern treatment.

## Background

Delayed presentation is a major problem contributing to the high burden and transmission of tuberculosis (TB) in most developing countries: Ethiopia, where fewer than half the estimated sputum smear positive pulmonary tuberculosis (PTB) cases are detected [1,2], is no exception. Nearly 10% of TB patients on treatment die each year from complications arising partly from delayed presentation and/or Human Immunodeficiency Virus (HIV) co-infection [1-3]. *Patient delay*, the time from onset of symptoms to first consultation to modern health care, is alarmingly prolonged with studies reporting from 2 to 4 months [4,5]. Patient delay represents 77% of the total delay period from onset of symptoms to initiation of treatment [6,7].

A key challenge of any TB control programme is to improve detection of sputum smear positive PTB cases. Normal practice is to use a passive case finding strategy. Patients' decisions on seeking health care however, depend on several factors, some common to many countries and settings, and some context-specific. In Ethiopia, inadequate awareness of TB, illiteracy, long distances to health services and the use of informal care from alternative providers have been associated with patient delay [4-6,8]. The HIV epidemic is counteracting TB control efforts by increasing TB incidence and contributing to delays in treatment seeking because of the stigma associated with HIV, and by association with TB [9]. Very little is known regarding the pattern of TB patients' health seeking behaviour from onset of symptoms to first consultation at a formal health facility. Identifying the sources, magnitude and risk factors of patient delay will help improve TB control. This cross sectional study was therefore developed to assess patterns of PTB patients' health seeking behaviour, the percent of patients who delayed seeking treatment and the risk factors for patient delays from onset of symptoms to seeking care at a public health facility, in Ethiopia.

## Methods

The study was carried out in Tigray Region, northern Ethiopia. The population of the region is estimated at 4.4 million, 86% of whom are rural. The region has five administrative zones comprising 36 districts. Public health institutions are the main sources of health care for most people: health services access, as defined by residence within 10 kilometres of any health institution, is about 65%. The TB control programme operates as an integral part of the public health system with control programme structures at national, regional and district levels. Patients have free access to TB diagnostic services and treatment in public health facilities. TB diagnostic services are only available in health centres and hospitals which are located at district urban centres. Rural patients presenting to clinics with symptoms suggestive of TB must therefore be referred to a health centre or a district hospital for diagnosis. Health centres can diagnose smear positive pulmonary TB using sputum microscopy, but must refer patients with negative sputum smears to district hospitals for further investigation. District hospitals are equipped with x-ray facilities and serve as the only diagnostic units for sputum smear negative PTB.

Our primary intention was to calculate a reliable estimate of the prevalence of patient delay in Tigray region. A common cut-off used to determine patient delay is 30 days. Previous reports from Ethiopia give crude estimates of the prevalence of patient delay as follows: 58% [4], 65% [8], 75% [5] and 79% [6]. To estimate the prevalence of patient delay with a 95% CI of width ± 5%, a sample size of 374 is needed, assuming a prevalence of 58% (i.e. the prevalence that gives the largest sample size) [10]. To improve the validity of our findings, we increased this sample size by a factor of 2.4 to allow for drop-out and to permit reliable estimates to be made for gender and other potential risk factors. To achieve this sample size, we randomly selected 10 districts from the total of 36 in Tigray region. Over a one year period, all newly diagnosed pulmonary TB patients ≥ 15 years of age from the study districts were recruited at the time of diagnosis. All suspected cases referred to the diagnostic centres (11 health centres and 10 hospitals) for diagnosis from all clinics (n = 48) in the study districts were included prospectively from January 12, 2005 to January 12, 2006. Ethical approval was obtained from the Tigray Regional Ethical Committee for Biomedical Research. Informed consent was obtained from each patient by interviewers (trained nurses).

Patients were interviewed using a pre-tested questionnaire translated into the local language regarding their health seeking behaviour and actions taken from onset of symptoms to first consultation. Patients were asked about the time of onset of cough and other major constitutional symptoms [11] and the types of treatment sought prior to first consultation at public health institutions. The number of days of treatment from informal sources was collected to quantify the magnitude of patient delays. Potential risk factors assessed included: socio-demographic factors (age, sex, marital status, literacy, area of residence), economic status (monthly income of household head, eligibility status for government food aid programme), health status (type of TB diagnosis, nutritional status, HIV sero-status), health seeking behaviour (use of alternative treatment/providers, type of health facility first visited, one way walking time to visit the first-visited health facility, how the decision was made to visit first-visited public health facility), perception and awareness of PTB (knowledge of cause of PTB, patient's perception of cause of his/her illness, patient's perception of potential risk of transmitting TB to family members, level of fear of revealing the disease to others). Patients' potential health access in terms of walking time to the nearest health facility was assessed in order to determine their pattern of consultation at public health facilities. Trained nurses collected blood samples for HIV testing, and took weight and height measurements for assessing their nutritional status at presentation. Laboratory technicians at diagnostic centres carried out anonymous HIV test (Determine^® ^HIV-1/2, Abbott Laboratories Limited, Illinois, USA).

Body Mass Index (BMI) was computed to determine the nutritional status of patients. Using the World Health Organization standard classification, patients were categorised as normal (BMI 18.5 to 24.99), moderate (BMI 17.5 to 18.49) and severely undernourished (BMI ≤ 17.5) [12]. In each district, patient records were crosschecked by two physicians to ascertain the accuracy of TB diagnosis. Generally, consultation is considered to be delayed if the first visit takes place more than three weeks after onset of symptoms [13]. However, in this study, to maintain comparability of findings with other studies [4,5,8] a patient delay between onset of constitutional symptoms and first consultation at a public health facility of ≥ 30 days was used as a cut off point. This is referred to subsequently as 'prolonged patient delay'.

Data entry and analysis was conducted with SPSS version 14 (SPSS, Inc., Chicago, USA). The percentage of patients who delayed seeking treatment and the median delays were calculated, and crude odds ratios and 95% confidence intervals were used to measure association between patients' demographic, economic, health status, perception and level of TB awareness and prolonged patient delay. Multivariate logistic regression analysis was used to estimate the independent effects (Adjusted odds ration, 95% confidence intervals) of each factor on prolonged patient delay. Variables with P-value ≤ 0.2 were entered into multivariate logistic regression models (stepwise) and allowing interaction for confounding effect between potential risk factors.

## Results

A total of 924 newly diagnosed PTB patients were prospectively enrolled from 21 diagnostic centres (10 hospitals and 11 health centres). Of these, 537 (58%) were sputum smear positive and 387 (42%) were sputum smear negative pulmonary cases. The median age was 34 years and 509 of the patients (55%) were above 29 years of age. Of the total of 924 patients, 413 (45%) were female, 576 (52%) were married, 451 (49%) were rural residents, 359 (39%) depended on farming for a livelihood and 430 (47%) were illiterate. Patients' median family size and the number of living rooms were 3 and 1 respectively.

Table 1 summarises patients' access to health care and health seeking behaviour. A total of 496 patients (54%) had not sought informal care for their symptoms between the onset of their illness and their first consultation at a public health facility, while 428 (46%) had sought care from at least one informal provider: 223 (24%) had been treated with holy water, 26 (3%) by traditional healers, 117 (13%) by private practitioners and 60 (6.5%) in private drug stores. 111 patients (12%) reported visiting at least two different informal providers prior to the first visit to a public health facility. The majority of patients (593 or 64%) made their first consultation at a district hospital, 231 (25%) attended a health centre and 100 (11%) a clinic. The median one way walking time from patient's residence to a public health facility was 40 minutes (with mean 68 minutes). Most patients (64%) decided to visit health facilities by themselves. Thirty-six percent of the patients visited a public health facility because they were advised to by members of their family, private practitioners or volunteer community health workers. Forty three percent of patients were accompanied by at least one person (with range 1 to 11 people) when they arrived at the public health facility.

**Table 1:** Health access and health seeking behaviour among pulmonary tuberculosis patients (n = 924) in Ethiopia during 2005–2006.

**Variables**	**Frequency (%))**
Treatment sought before the first visit at public health facilities:	
No prior treatment	496 (54)
Holy water	223 (24)
Private practitioners	117 (13)
Private drug stores/pharmacies	62 (7)
Traditional healers	26 (3)
The number of visits made at alternative treatment/provider:	
None	496 (54)
One	316 (34)
Two	91 (10)
Three	17 (2)
Four	3 (0.3)
Type of public health facilities visited at first consultation:	
Clinics	100 (11)
Health centres	231 (25)
Hospitals	593 (64)
One way walking time to health facilities:	
≤ 40 minutes (median)	469 (51)
> 40 minutes	455 (49)
Who made the decision to visit public health facilities?	
Patients themselves	594 (64)
Family members	210 (23)
Health workers (private practitioners)	42 (5)
Others^1^	78 (8)

^1 ^Volunteer community health workers, neighbours and friends.

The median patient delay among those who had used informal treatment was 31 days (Table 2). Patients with no prior history of informal treatment had the lowest patient delay (median = 15 days). The longest patient delay (median = 90 days) recorded was among patients who had been treated with holy water. The overall total median and mean patient delays were 30 and 60 days respectively. Of the total patients, 53%; 95% CI (50%, 56%) had delayed consultation for 30 days and longer. Patient delay was longer than 90 days in 15% of cases. Patient delay among women was 54%; 95% CI (54%, 58%) and men 51%; 95% CI (47%, 55%).

**Table 2:** Sources of patient delays among pulmonary tuberculosis patients (n = 924) in Ethiopia during 2005–2006.

		**Days of delay**	
		
**Sources of alternative treatment**	**N (%)**	**Mean**	**Median (25–75 percentiles)**
Use of alternative treatment:			
Holy water	223(24)	92	90 (28 – 90)
Private practitioners	117(13)	63	30 (10 – 60)
Private drug stores/pharmacies	62 (7)	63	30 (14 – 90)
Traditional healers	26 (3)	65	30 (18 – 81)
Sub-total	428(46)	79	31 (15 – 90)

No prior use of alternative treatment	496(54)	45	15 (7 – 49)

Total patient delay	924(100)	60	30 (7 – 60)

Univariate analyses of risk factors for prolonged delay (first consultation after ≥ 30 days of onset of symptoms) are shown in Tables 3 to 5. Prolonged patient delay was significantly associated with literacy, rural residence, type of TB disease, occupation, nutritional status and whether patients were on government food aid programme. No significant association was found between prolonged patient delay and sex, age, marital status, HIV status and monthly income (Table 3). There was an association between prolonged patient delay and use of informal treatment prior to first consultation at public health facilities, the type of health facilities first visited by patients and longer one way walking time to a nearby health facility (Table 4). Prolonged patient delay was not related with patients' awareness of the cause of PTB, perception of risk of transmitting the disease to family members or the level of fear to reveal their disease to others (Table 5).

**Table 3:** Demographic, economic, health status of pulmonary tuberculosis patients (n = 924) and patient delayed first consultation at public health facilities in Ethiopia during 2005–2006.

	**Patient delay**			
			
**Variables**	≥ 30 days n (%)	< 30 days n (%)	**COR (95% CI)^1^**	**P-value**
**Sex:**				
Male	260 (51)	251(49)	1	
Female	233(56)	180 (44)	1.25 (0.9, 1.6)	0.09
**Age:**				
15–29	219(53)	196(47)	1	
> = 30	274(54)	235(46)	1.04 (0.8,1.3)	0.7
**Education**				
Illiterate	267(62)	163(38)	2.5 (1.9, 3.5)	0.0001
Elementary	111(55)	89(45)	1.9 (1.3, 2.8)	0.0001
Secondary	115(39)	179(61)	1	
**Residence**				
Urban	216(46)	257(54)	1	
Rural	277(61)	174(39)	1.9 (1.45, 2.5)	0.0001
**Occupation**				
Farming	220(61)	139(39)	1	
Employed	81(45)	101(55)	0.51 (0.35, 0.7)	0.0001
Daily labourer	72(49)	76(51)	0.6 (0.4, 0.8)	0.009
Dependents	120(51)	115(49)	0.66 (0.5, 0.9)	0.01
**Marital status:**				
Married	265(56)	211(44)	1	
Single	162(52)	152(48)	0.85 (0.6, 1.1)	0.2
Divorced/widowed	66(49)	68(51)	0.77 (0.52, 1.13)	0.1

**HIV status**				
Positive	215 (52)	197 (48)	1	
Negative	278(54)	234(46)	1.1 (0.8, 1.4)	0.5
**Nutritional status **^2^				
Normal	97 (49)	101(51)	1	
Moderate	56 (44)	70 (56)	0.83 (0.53, 1.3)	0.06
Severe malnutrition	340 (57)	260 (43)	1.4 (0.98, 1.8)	0.43
**Type of pulmonary disease**				
Sputum smear negative	185 (48)	202 (52)	1	
Sputum smear positive	308 (57)	229 (43)	1.5 (1.2, 1.9)	0.004

**Monthly income **^3^				
<100 Birr	329 (54)	276 (46)	1	
101–200 Birr	102 (58)	75 (42)	1.1 (0.8, 1.6)	0.4
≥ 201 Birr	62 (44)	80 (56)	0.65 (0.5, 0.9)	0.02
**Receive food aid from the government:**				
No	353(51)	340 (49)	1	
Yes	140(61)	91(39)	1.5 (1.2, 2)	0.01

^1 ^Crude Odds Ratio (95% Confidence Interval. ^2 ^Measured using body mass index. ^3 ^Nine Birr = 1$.

**Table 4:** Health access and health care seeking characteristics of pulmonary tuberculosis patients (n = 924) and patient delay at first consultation in Ethiopia during 2005–2006.

	**Patient delay**			
			
**Variables**	≥ 30 days n (%)	< 30 days n (%)	**COR (95% CI)**	**P-value**
Treatment sought prior to first visit in public health facilities:				
No prior treatment	211(43)	285(57)	1	
Holy water	166(74)	57(26)	3.9(2.8, 5.5)	0001
Traditional healers	18(61)	10(39)	2.2(0.9, 4.8)	0.06
Private practitioners	64(55)	53(45)	1.6(1.1, 2.4)	0.02
Private drug stores/pharmacies	34(57)	26(43)	1.8(1.1, 3)	0.04
The number of alternative treatments sought prior to the first consultation				
Zero	212(43)	285(57)	1	
One	199(63)	117(37)	2.8(1.7, 3)	0001
Two	65(71)	26(29)	3.4(2, 5.4)	0.002
Three	14(82)	3(18)	6.3(1.7, 77)	0.004
Four times	3	-	-	-
Type of health facilities visited for the first time since onset of illness:				
Hospitals	297(50)	296(50)	1	
Health centres	131(57)	100(43)	1.3(0.9, 1.8)	0.08
Clinics	65(65)	35(35)	1.9(1.2, 2.8)	0.006
One way walking time to first visited health facility:				
≤ 1 hour (median)	226(48)	243(52)	1	
> 1 hour	267(59)	188(41)	1.5(1.2, 1.9)	0.001
Decision to visit the first public health facilities was made by:				
Patients themselves	308(52)	286(48)	1	
Private practitioners	23(55)	19(45)	1.1(0.6, 2.1)	0.7
Family members	126(60)	84(40)	1.4(1.01,1.9	0.04
Others^1^	36(46)	42(54)	0.8(0.5,1.5)	0.3

^1 ^Volunteer community health workers, neighbours and friends.

**Table 5:** Knowledge and perception of tuberculosis and status of patient delay at first consultation among pulmonary tuberculosis patients (n = 924) in Ethiopia during 2005–2006.

	**Patient delay**			
			
**Variables**	≥ 30 days n (%)	<30 days n (%)	**COR (95% CI)**	**P-value**
**Ever heard of PTB**				
Yes	433(52)	402(48)	1	
No	59(69)	27(31)	0.49(0.31, 0.79)	0.004
**Patients' awareness about the main cause of PTB**				
Contact with another patient	60(38)	97(62)	1	
Evil spirit, gods will, other ^1^	64(63)	37(37)	2.8(1.6, 4.6)	0.0001
Exposure to cold	260(53)	235(47)	1.8(1.2, 2.5)	0.002
I do not know	109(64)	62(36)	2.7(1.8, 4.4)	0.0001
**Patients' perception bout the main cause of their illness**				
Contact with a TB patient	71(47)	79(53)	1	
Evil, gods' will and other ^1^	48(65)	26(35)	2.1(1.15, 3.6)	0.014
Exposure to cold	234(52)	216(48)	1.2 (0.84, 1.7)	0.3
Inherited, malnutrition and sexually	46(49)	48(51)	1.1(0.65, 1.78)	0.7
I do not know	94(60)	62(40)	1.7(1.1, 2.6)	0.024
**Do you think that you could potentially transmitting TB to your family?**				
Yes	425(53)	377(47)	1	
No	68(56)	54(44)	1.1 (0.7, 1.5)	0.5
**Would you be worried if others know about your disease?**				
No	431(53)	381(47)	1	
Yes	62(55)	50(45)	1.1(0.73,1.6)	0.6

^1 ^Exhaustion and eating certain types of food

Table 6 shows the results of the multivariate analysis of risk factors for prolonged patient delay. Prolonged delay was significantly higher among illiterate patients than those who had completed secondary school [AOR 1.7; 95% CI (1.2 to 2.4); p = 0.004]. Rural patients were more likely to have prolonged delay than urban patients [AOR 1.4; 95% CI (1.1 to 1.9); p = 0.02]. The risk of prolonged delay was significantly higher for sputum smear positive pulmonary patients than sputum smear negative pulmonary patients [AOR 1.4; 95% CI (1.1 to 1.9); p = 0.02]. The use of prior treatment with holy water [AOR 3.5; 95% CI (2.4 to 5); p = 0.0001], by private practitioners [AOR 1.7; 95% CI (1.1 to 2.6); p = 0.01] and drug stores [AOR 1.9; 95% CI (1.1 to 3.5); p = 0.002] was significantly associated with prolonged delay. Patients' lack of awareness about the cause of PTB [AOR 1.6; 95% CI (1.1 to 2.7); p = 0.05] or poor perception of the cause of TB such as evil [AOR 2; 95% CI (1.2 to 3.8); p = 0.01] were related a higher risk of prolonged delay.

**Table 6:** Multivariate analysis of risk factors associated with prolonged patient delay among pulmonary tuberculosis patients (n = 924) in Ethiopia during 2005–2006.

**Factors**	**Crude Odds Ratio** **(95% CI)**	**Adjusted Odds Ratio** **(95% CI)**	**P-value**
**Female sex**	1.2 (0.9, 1.6)	1.4 (0.9, 1.8)	0.06
**Education**			
Completed secondary	1	1	
Completed elementary	2.5 (1.9, 3.5)	1.1 (0.77, 1.6)	0.6
Illiterates	1.9(1.3, 2.8)	1.7 (1.2, 2.4)	0.004
**Rural residence**	1.9(1.45, 2.5)	1.4 (1.1, 1.9)	0.02
**Marital status**			
Married	1	1	
Single	0.85(0.6, 1.1)	1.1 (0.82, 1.6)	0.4
Divorced/widowed	0.77(0.52, 1.13)	0.67(0.44, 1.02)	0.06
**Type of disease:**			
Sputum smear negative	1	1	
Sputum smear positive	1.5 (1.2, 1.9)	1.4 (1.1, 1.9)	0.02
**Use of alternative treatment before first consultation:**			
No prior treatment	1	1	
Holy water	3.9(2.8, 5.5)	3.5 (2.4, 5)	0.0001
Traditional healer	2.2(0.9, 4.8)	2 (0.9, 4.5)	0.1
Private practitioner	1.6(1.1, 2.4)	1.7 (1.14, 2.6)	0.01
Private drug stores	1.8(1.03, 3)	1.9 (1.1, 3.5)	002
**Patients' perception about the cause of their illness:**			
Contact from PTB patient	1	1	
Evil or bad lack	2.1(1.15, 3.6)	2 (1.2, 3.8)	0.01
Exposure to cold	1.2 (0.84, 1.7)	1.4 (0.95, 2.1)	0.08
I do not know	1.1(0.65, 1.78)	1.6 (1.1, 2.8)	0.05

## Discussion

Our finding shows that patient delay in Ethiopia is longer than the recommended three weeks for detecting a suspected TB case [8], with 53% of patients delaying their first consultation at a public health facility for 30 days or more. The median delay among patients who sought treatment from informal sources (31 days) was higher than those who did not (15 days). Patients who have been treated with holy water and by private practitioners contributed 52% and 27% of the total delayed consultations respectively. The overall median patient delay of 30 days was consistent with previous studies from Ethiopia [4-6,8] and other African countries [14,15].

Several factors were found to be associated with prolonged patient delay in the study districts. Prolonged patient delay has been shown to be more common among women than men by African studies [16,17]. Our study however did not show a significant association between sex and prolonged patient delay, a finding consistent with previous Ethiopian studies [5,6,8]. More male than female (55% versus 45%) cases were detected over the study period, but no clear explanation for this arose. Elsewhere [18], similar differences in case detection have been attributed to women's limited decision-making power and failure of health systems to provide accessible and acceptable health care: others suggest the difference is due to differences in biological susceptibility between men and women [19]. Further investigations are needed in this area.

In line with previous reports [5,6], illiteracy and rural residence were significantly associated with prolonged patient delay. Neither low monthly income nor dependence on government food aid programme was an independent predictor of prolonged patient delay. The lack of association of low income with prolonged delay could be due to its interaction with patients' area of residence and other economic parameters used in this study. Based on the parameters used to measure patients' economic status, most (66%) were destitute (earning less than 1 United States dollar per day) [20] and 25% were on government food aid programme targeted to support households with no means of livelihood. In Ethiopia, patients are entitled to free medical care in public health facilities when they have been ascertained unable to pay. Of our respondents, 239 (26%) were entitled to free medical care while 345 (38%) failed to qualify and 324 (36%) did not ask for such services. This shows that more than a third of patients were unable to pay even the minimal medical consultation fee seen in Ethiopia (US$ 0.2 for the first consultation). These findings indicate that rural residence could be an appropriate proxy measure of poverty in Ethiopia and the high level of rural poverty may preclude equal proportions of rural and urban patients [AOR = 1.43; 95%CI (1.1 to 1.9); p = 0.02] from seeking timely care in public health facilities. Being a rural resident has been reported to be a strong marker of poverty and an independent predictor of patient-delayed first consultation in other studies in Ethiopia [8].

### Health seeking behaviour

The use of an informal treatment/provider was a strong predictor of prolonged patient delay in this study. Endogenous traditional medicine is widely practiced in Ethiopia [21]. Patient delayed consultation for modern health care has been associated with the use of traditional medicine in Ethiopia [4,6,8] and in sub-Saharan Africa [16,22]. However, the relative contribution of such alternative sources of treatment to patient delay has never been thoroughly investigated. Our findings indicate that 46% of patients had sought modern care after informal treatment failed. In the order of their contribution to patient delay, the following alternative sources of treatment were identified: holy water from orthodox churches, private practitioners, traditional healers and private drug vendors/pharmacies.

The Orthodox Church, the most prominent religious institution in Ethiopia, widely practices faith-based therapy with holy water. Patients get holy water treatment both at home and in designated monasteries/churches with springs believed to have supernatural powers including healing. The home-based treatment is prescribed by priests after sanctifying a small volume of water to be applied over the body of the diseased and sprayed inside his/her living room. An alternative approach demands patients' stay close to holy water springs that are mostly located adjoining to churches/monasteries representing different saints. The choice and duration of treatment depends on patients' beliefs about a particular saint and priests' recommendations. In the study region, there are over 5000 Orthodox Churches with nearly 20,000 priests giving services. Followers of Orthodox Christianity constitute 95% of the total regional population. The use of holy waters as an initial treatment will probably continue to be a cause of patient delayed presentation in areas where public health facilities are remotely located.

In Ethiopia, the TB control programme does not extend into the formal private health sector. In this study, 13% of patients visited private practitioners prior to first consultation at a public health facility. More urban than rural (17% versus 10%) patients made their first consultation at a private practitioner. Smaller proportions of patients consulted private drug vendors and traditional healers (7% and 3% respectively). The involvement of these primary sources of care in TB control will be crucial in addressing the prolonged patient delay in Ethiopia. We therefore suggest that public-private partnership [23] may play a significant role in the early referral and treatment of pulmonary patients. The involvement of these institutions in TB control could be a feasible strategy to reduce prolonged patient delays in urban areas.

**Figure 1:**
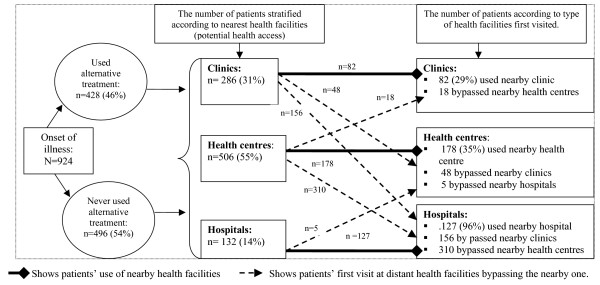
**Health seeking behaviour of pulmonary tuberculosis patients (n = 924) according to attendance at alternative providers and public health facilities in Ethiopia during 2005–2006**.

In contrast to previous reports [4,24,25], poor access to health care in terms of travel time from patients' areas of residences to public health facilities was not significantly associated with prolonged patient delay. After prolonged delay, the pattern of utilization of public health facilities was found to be complex. In this study, a majority of patients bypassed nearby facilities in favour of a district diagnostic centre. More than half of rural patients bypassed nearby clinics and made their first consultation at a district diagnostic centre (a hospital or health centre). Similarly, more than half of the patients whose residence was closer to a health centre still made their first consultation at district hospitals. These attendance patterns are summarised in Figure 1. These findings suggest that proximity in terms of walking time to health facilities alone may not improve patients' health seeking behaviour for early TB treatment. This could be due to the fact that most patients consult after their health status is compromised and getting optimal quality of care from tertiary facilities could be a priority to regain their ability to function normally.

### Perception and stigma

Most patients had heard of PTB, but only a few cited the infectious nature of the disease. The majority of patients believed TB to be the result of exposure to cold, evil spirits and God's will. In line with previous studies [4,6,8], patients with poor perception about TB were more likely to delay their first consultation at public health facilities, suggesting that renewed efforts are required to improve awareness of TB.

The stigma attached with TB often deters patients from seeking early treatment [2,24]. Nonetheless, most of the patients (88%) were willing to reveal their illness to others. Only a few patients (12%) preferred not to reveal their illness. Fear of isolation (8%) and lost hope of being cured (4%) were given as explanations. With the spread of HIV, TB-related stigma has been heightened in sub-Saharan Africa [26,27]. In Ethiopia, the burden of TB is still increasing with the spread of HIV from urban to rural areas. Earlier studies estimate that HIV co-infection rate in TB patients varies from 20% to 47% [28-30]. Nevertheless, estimates from these studies could be unreliable since most were conducted in major urban centres. The NTC programme HIV surveillance report indicates that 40% of PTB patients were HIV positive [31]. In this study, 45% of respondents were HIV sero-positive. This was consistent with the national TB programme figures reported for years 2005/2006 [31]. However, only 8% of patients thought that their illness was associated with HIV. This signifies that most respondents had low index of suspicion about the risk of having HIV. These results are consistent with a recent survey from the Southern region of Ethiopia where only 7% patients equated the two diagnoses [8]. Improving awareness about TB and its treatment, and the stigma attached with TB/HIV are crucial to reduce prolonged delays in seeking modern TB care.

### Limitations of the study

The low specificity of the diagnostic methods (radiography and clinical features) used to detect sputum smear negative PTB could potentially lead to misdiagnosis of patients. Because of atypical clinical presentations among cases with HIV co-infection, the probability of introducing bias could be high. The other potential limitation may arise from patients' inability to recall the exact date of onset of symptoms based on which the study outcomes were measured. However, measures were taken to minimize these two limitations. The diagnosis of sputum smear negative PTB cases has been made by experienced clinicians using the recommended diagnostic algorisms [13]. In each district, two clinicians also reviewed records in order to validate patients' history of illness at presentation with their responses from interview, and to evaluate whether patients were diagnosed as per the national diagnostic standard [11]. Patients were interviewed by experienced nurses regarding date of onset of cough and other constitutional symptoms. To minimize variations in estimating patient delay, interviewers used major national and local events to define patients' perceived date of onset of symptoms.

This study included all patients attending health facilities in the study districts during the study period. That is, this study was of self-referent patients and it is unknown whether there are differences in patients' characteristics with the non-attendant cases. The implications of this on the findings, however, are likely to be limited for a number of reasons. The number of PTB cases recruited constitutes 60% of the total expected adult cases for these districts. The number recruited was 2.4 times the required study size for this study. TB care is only available in public health facilities in these districts (and in Ethiopia in general), and have no where else to go. This study was conducted within operational conditions of the TB control programme. The study approach is consistent with the WHO Stop TB strategy and national TB programme policy.

## Conclusion

The high occurrence of delayed consultation among PTB patients a major challenge for TB control programme in Ethiopia. Illiteracy, rural poverty, poor perception of TB and the use of alternative treatment are key factors contributing to delays in seeking modern medical treatment. Delay in seeking allopathic treatment among sputum smear positive PTB patients is a major constraint to curtailing the continued transmission of TB. Creating public awareness and the involvement of the private health sector, traditional practitioners and religious institutions in TB control could be important in order to improve early detection of PTB. The findings of the study could also be relevant to other sub-Saharan countries where poverty, illiteracy, HIV and the use of traditional treatment for TB disease are highly prevalent.

## Competing interests

The authors declare that they have no competing interests.

## Authors' contributions

MMM, RJM, and AG participated in the conception and design of the study. MMM, RJM and AG monitored and supervised data collection. MMM, JNN and JDW managed the data, did the analysis and interpretation, and wrote the first and final draft of the manuscript. The manuscript was substantially revised by each author. All authors read and approved the final manuscript.

## Pre-publication history

The pre-publication history for this paper can be accessed here:


